# GLTSCR2 is an upstream negative regulator of nucleophosmin in cervical cancer

**DOI:** 10.1111/jcmm.12474

**Published:** 2015-03-27

**Authors:** Jee-Youn Kim, Young-Eun Cho, Yong-Min An, Sang-Hoon Kim, Yong-Gwan Lee, Jae-Hoon Park, Sun Lee

**Affiliations:** Department of Pathology, College of Medicine, Kyung Hee UniversitySeoul, Korea

**Keywords:** GLTSCR2, nucleophosmin, cervical cancer, nucleoplasmic translocation

## Abstract

Nucleophosmin (NPM)/B23, a multifunctional nucleolar phosphoprotein, plays an important role in ribosome biogenesis, cell cycle regulation, apoptosis and cancer pathogenesis. The role of NPM in cells is determined by several factors, including total expression level, oligomerization or phosphorylation status, and subcellular localization. In the nucleolus, NPM participates in rRNA maturation to enhance ribosomal biogenesis. Consistent with this finding, NPM expression is increased in rapidly proliferating cells and many types of human cancers. In response to ribosomal stress, NPM is redistributed to the nucleoplasm, where it inactivates mouse double minute 2 homologue to stabilize p53 and inhibit cell cycle progression. These observations indicate that nucleolus-nucleoplasmic mobilization of NPM is one of the key molecular mechanisms that determine the role of NPM within the cell. However, the regulatory molecule(s) that control(s) NPM stability and subcellular localization, crucial to the pluripotency of intercellular NPM, remain(s) unidentified. In this study, we showed that nucleolar protein GLTSCR2/Pict-1 induced nucleoplasmic translocation and enhanced the degradation of NPM *via* the proteasomal polyubiquitination pathway. In addition, we showed that GLTSCR2 expression decreased the transforming activity of cells mediated by NPM and that the expression of NPM is reciprocally related to that of GLTSCR2 in cervical cancer tissue. In this study, we demonstrated that GLTSCR2 is an upstream negative regulator of NPM.

## Introduction

Nucleophosmin (NPM)/B23, a multifunctional nucleolar protein, plays an important role in key cellular processes such as ribosome biogenesis 1, apoptosis 2 and cell proliferation 3, as well as in pathological conditions including cancer development or progression 4. NPM is overexpressed in many types of human cancers 5 and is one of the most frequent sites of genetic alteration in haematopoietic tumours 6; chromosomal translocation of NPM gene with or without unusual cytoplasmic expression of NPM protein is reported to be associated with development of several forms of leukaemias or lymphomas 7,8. In addition, nuclear distribution of NPM is reported to be an important determinant of its role in oncogenic stress, including DNA damage. For example, NPM forms protein complexes with alternative reading frame (ARF), a positive regulator of p53, and traps it within the nucleolus to prevent p53 activation 9. However, in response to DNA damage or ribotoxic stress, NPM is redistributed into the nucleoplasm, where it inactivates mouse double minute 2 (MDM2) to stabilize p53 and inhibit cell cycle progression 10,11. These findings indicate that regulation of the nucleoplasmic translocation process as well as total expression level and genetic alteration of NPM comprise the key mechanisms of the oncogenic activity of NPM. Nevertheless, the upstream molecule(s) regulating the subcellular localization and expression levels of NPM remain to be identified.

Glioblastoma tumour suppressive candidate region gene 2 (GLTSCR2)/Pict-1 is a nucleolar protein with multiplex activity in both, oncogenesis and tumour suppression 12–14. The expression of GLTSCR2 is down-regulated in several human cancers, including brain and ovarian tumours 13–15, but increases in gastric and oesophageal cancers 12. In the nucleolus, GLTSCR2 captures ribosomal protein (RP) L11 (RPL11) to prevent its nucleoplasmic translocation, which in turn suppresses MDM2-mediated degradation of p53. In contrast, nucleoplasmic redistribution of GLTSCR2 allows for direct interaction with p53, which stabilizes the protein 16. These results suggest that both, expression levels and subnuclear localization of GLTSCR2 contribute to p53-dependent tumour suppressive activity. However, the molecular mechanisms of GLTSCR2 activity on cancer development or progression are largely unknown.

The purpose of this study was to investigate the role of GLTSCR2 in regulating the oncogenic activity of NPM in human cervical cancer. In this report, we describe that GLTSCR2 is a negative regulator of NPM, in that GLTSCR2 enhances NPM degradation through the proteasomal polyubiquitination pathway and decreases the transforming activity induced by NPM overexpression. Our results imply that GLTSCR2 may be a therapeutic target for cancer treatment *via* NPM dysregulation.

## Materials and methods

### Cell lines, cell cultures, tissue samples and reagents

HeLa uterine cervix cancer cells were obtained from the American Type Culture Collection (Rockville, MD, USA). Cells were cultured in DMEM with 10% foetal bovine serum (FBS) and penicillin-streptomycin (Gibco, Grand Island, NY, USA) in a humidified incubator. A total of 52 human cervical cancer samples and 24 normal cervical tissue samples in formalin-fixed and paraffin-embedded tissue blocks were collected using an Institutional Review Board-approved protocol. The rabbit polyclonal antibody against GLTSCR2 was prepared as described previously 17. Mouse anti-NPM antibody was purchased commercially from Cell Signaling Technology, Inc. (Danvers, MA, USA). Unless otherwise specified, all other reagents were obtained from Sigma-Aldrich, Inc. (St Louis, MO, USA).

### Plasmid construction, transfection and GLTSCR2 knockdown

Plasmids for wild-type or mutant forms of GLTSCR2 and NPM were generated using polymerase chain reaction and standard cloning techniques as previously described 17. Cells were transfected with plasmids using Lipofectamine 2000 (Invitrogen, Carlsbad, CA, USA) according to the manufacturer's recommendations. The expression of GLTSCR2 was knocked down by transfecting siRNA targeted at GLTSCR2 (Qiagen Inc., Valencia, CA, USA) using oligofectamine (Invitrogen).

### Soft agar colony-forming assay

Equal volumes of 2× RPMI with 20% FBS and 1% melted agar were mixed at 40°C and added to 60-mm plates to form the base agar layer. After trypsinizing the cells, 10^4^ HeLa cells (singly isolated) were suspended in 2× RPMI with 20% FBS and then mixed well with an equal volume of 0.7% melted agar at 40°C. The cell suspension was then added quickly to the plate onto the solidified base agar layer. The plates were incubated at 37°C in a humidified CO_2_ incubator for 3 weeks and 0.5 ml of cell culture medium was added twice a week to nourish the cells. After 3 weeks, the plates were stained with 0.005% crystal violet for 1 hr, washed with distilled water, and the colonies were counted under a light microscope.

### Western blot, immunocytochemistry, immunohistochemistry (IHC) and evaluation of IHC

Western blot and immunocytochemical staining were performed as previously described 17. Immunohistochemical staining of cervical cancer and normal mucosal tissues for GLTSCR2 and NPM was also performed and evaluated as previously described 18.

### Ubiquitination assay

His-tagged ubiquitin and plasmids expressing either GLTSCR2 or NPM were transfected into HeLa cells for 48 hrs and then cells were treated with MG132 (20 μM) for 6 hrs to inhibit proteasomal degradation. Cells were harvested in lysis buffer [6 M guanidine-HCl, 0.1 M NaPi, 10 mM Tris-HCl (pH 8.0), 5 mM imidazole, 10 mM β-mercaptoethanol] and treated with Ni^2+^-chelating Sepharose for 3 hrs at room temperature, then washed five times with buffer solution [8 M urea, 0.1 M NaPi, 10 mM Tris-HCl (pH 6.3), 10 mM β-mercaptoethanol]. Western blot was performed, followed by anti-GFP antibody staining for NPM.

### Statistical analysis

Statistical analysis was carried out using SPSS software, version 13.0 (SPSS, Chicago, IL, USA). Data were analysed with Fisher exact test or a Pearson chi-squared test. The correlations between GLTSCR2 and MPM expressions were determined by Spearman's rank correlation. Differences were considered statistically significant when *P* < 0.05.

## Results

### GLTSCR2 regulates nucleoplasmic translocation of nucleolar NPM

**Figure 1:**
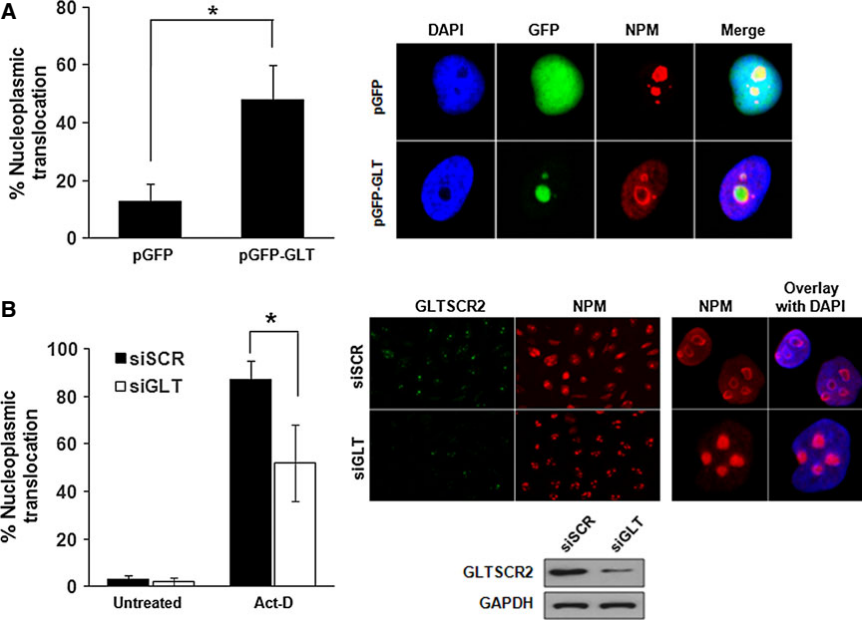
GLTSCR2 induces nucleoplasmic translocation of nucleolar NPM. (A) HeLa cells were transfected with 2 μg pGFP or pGFP-GLT plasmid and immunostained with anti-NPM antibody after 48 hrs of transfection. Cells with nucleoplasmic translocation of NPM were counted among at least 100 GLTSCR2-transfected cells under a fluorescence microscope (left panel). Data from three independent experiments are shown as mean ± SD; *P < 0.05. Representative images were shown in the right panel (original magnification, ×600). (B) HeLa cells transfected with scrambled siRNA (siSCR) or siRNA targeted at GLTSCR2 (siGLT) for 72 hrs were treated with 50 μM Act-D for 2 hrs or left untreated. Then, cells with nucleoplasmic translocation of NPM were counted among at least 200 cells after co-immunostaining with anti-GLTSCR2 and anti-NPM antibodies (left panel). Data (mean ± SD) are the results of three independent experiments. *P < 0.05. Representative immunofluorescence images are shown in the middle (original magnification, ×200) and right (original magnification, ×400) upper panels. Cell lysates transfected with siSCR or siGLT for 48 hrs were Western blotted to detect GLTSCR2 expression and images are shown in the right lower panel. GAPDH was used as the loading control.

Most of the nuclear content of proteins GLTSCR2 and NPM, is in the nucleolus, however they translocate to the nucleoplasm in response to oncogenic stress, such as DNA damage, where they inhibit cell cycle progression 19,20. Therefore, we initially investigated whether overexpression or down-regulation of GLTSCR2 affected nucleolar NPM translocation. HeLa cells were transfected with mock plasmid (pGFP) or GFP-tagged GLTSCR2-expressing plasmid (pGFP-GLT) for 48 hrs and stained with anti-NPM antibody to determine the intranuclear localization of NPM. As shown in Figure1A, significant translocation of nucleolar NPM into the nucleoplasm was noted in pGFP-GLT-transfected cells, but not in pGFP-transfected cells. Next, to elucidate the effects of GLTSCR2 down-regulation on NPM translocation, HeLa cells were transfected with either siRNA targeted at GLTSCR2 (siGLT) or scrambled siRNA (siSCR). After 72 hrs of transfection, cells were treated with 50 μM actinomycin D (Act-D) for 2 hrs and NPM localization analysis was performed. As shown in Figure1B, GLTSCR2 down-regulation significantly decreased the Act-D-induced nucleoplasmic translocation of nucleolar NPM. Together, our results demonstrated that GLTSCR2 regulated the nucleoplasmic mobilization of NPM.

### GLTSCR2 down-regulates total NPM expression levels by decreasing its protein stability

**Figure 2:**
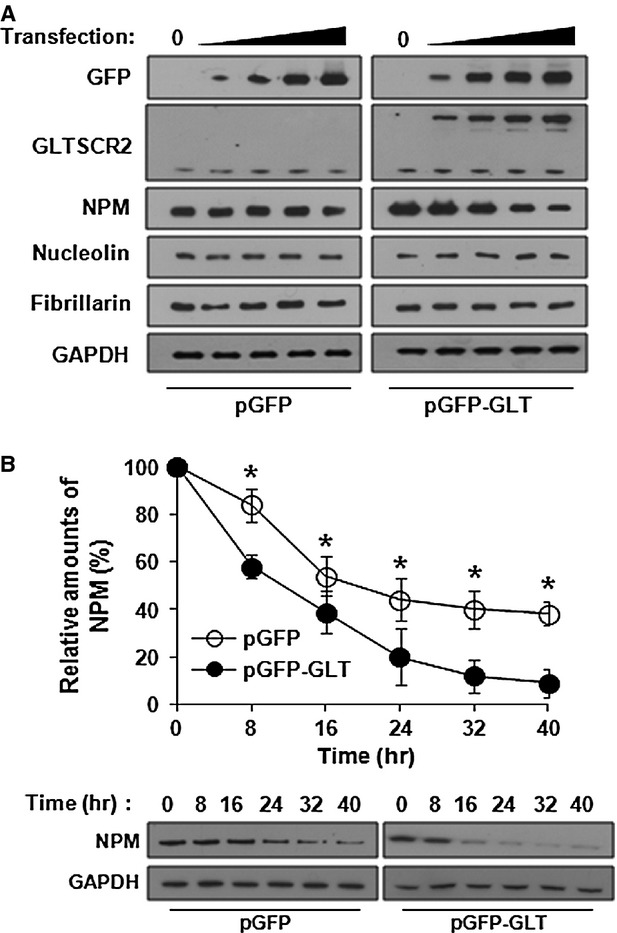
GLTSCR2 down-regulates NPM expression. (A) HeLa cells were transfected with increasing amounts (0, 1, 2, 3 or 4 μg/10^6^ cells) of pGFP or pGFP-GLT for 48 hrs and cell lysates were Western blotted using indicated antibodies. (B) HeLa cells were transfected with pGFP-GLT or pGFP-GLT for 24 hrs. After treatment with 100 μg/ml cycloheximide, cell lysates were prepared at 8-hr intervals from 0 to 40 hrs. The amount of NPM was determined by Western blot after normalization with GAPDH. The plot shown is the densitometric quantification of cycloheximide chase assay (upper panel). Data represent percentages of NPM intensity of three independent experiments compared with that at the zero time-point (mean ± SD; *P < 0.05). Representative Western blot images of cycloheximide chase assay are shown in the lower panel.

We investigated whether GLTSCR2 regulated the expression levels of NPM in addition to translocation. HeLa cells were transfected with increasing amounts of either pGFP or pGFP-GLT, and NPM expression levels were determined after 48 hrs of transfection. As shown in Figure2A, ectopic expression of GLTSCR2 down-regulated the expression of NPM in a GLTSCR2-dependent manner, whereas mock transfection did not change the NPM expression levels. In addition, GLTSCR2-induced down-regulation was specific to NPM, as opposed to other nucleolar stress-response proteins, as ectopic GLTSCR2 expression did not alter the expression levels of nucleolin and fibrillarin (Fig.2A). To elucidate the mechanism of NPM down-regulation by GLTSCR2, we examined the protein stability of NPM by cycloheximide chase assay. HeLa cells were treated with cycloheximide and the half-life of NPM was determined using densitometric analysis. The half-life was 24 hrs in pGFP-transfected cells but was reduced to 10 hrs in pGFP-GLT-transfected cells (Fig.2B). However, NPM mRNA levels remained constant after pGFP or pGFP-GLT transfection (data not shown). Taken together, our results demonstrated that GLTSCR2 promoted degradation of NPM.

### GLTSCR2 enhances NPM degradation through proteasomal ubiquitination pathway

**Figure 3:**
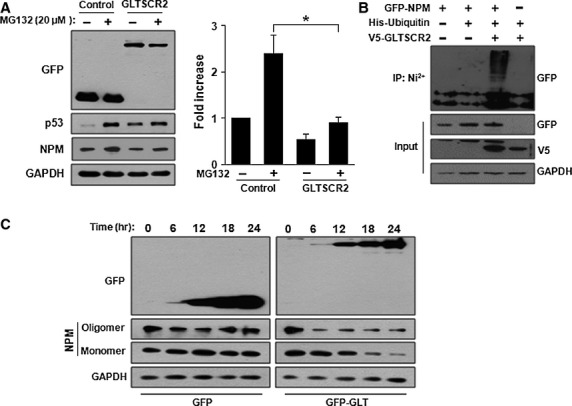
GLTSCR2 enhances NPM degradation through proteasomal ubiquitination pathway. (A) HeLa cells were transfected with pGFP (control) or pGFP-GLT (GLTSCR2) for 48 hrs. Cells were either not treated or treated with 20 μM MG132 for 6 hrs and cell lysates were Western blotted using the indicated antibodies (left panel). The histogram plot shown is the densitometric quantification of NPM after normalization to MG132-untreated pGFP-transfected cells (mean ± SD; *P < 0.05). (B) HeLa cells were transfected with the indicated plasmids. Cells were further treated with 20 μM MG132 for 6 hrs and lysates were pulled down by Ni^2+^-chelating Sepharose for 3 hrs. The resultant pellets were Western blotted using anti-GFP antibody (upper panel). The expression levels of GFP-NPM, V5-GLTSCR2 and GAPDH are shown in the three lower panels. (C) GLTSCR2 suppresses oligodimerization of NPM. HeLa cells were transfected with pGFP or pGFP-GLT for 24 hrs. Then, cells were treated with 0.1% glutaraldehyde and lysates were Western blotted to detect monomeric or oligomeric forms of NPM after normalization with GAPDH.

Nucleophosmin is degraded through the 26S proteasomal polyubiquitination pathway 21. Therefore, to investigate whether GLTSCR2 enhances NPM degradation through the proteasomal pathway, we treated pGFP-GLT-transfected HeLa cells with the 26S proteasome inhibitor MG132 and compared the degradation of NPM with pGFP-transfected cells used as control. Treatment with 20 μM MG132 for 6 hrs stabilized endogenous p53 levels as compared to untreated cells, indicating an efficient blockage of the 26S proteasomal degradation pathway (Fig.3A). Under the same conditions of MG132 treatment, the majority of NPM degradation induced by GLTSCR2 was blocked (Fig.3A), demonstrating that NPM degradation by GLTSCR2 is dependent on the function of the 26S proteasomal pathway. Next, we performed ubiquitination assays for NPM to investigate whether GLTSCR2 affects polyubiquitination of NPM. HeLa cells were transfected with a plasmid expressing GFP-tagged NPM (GFP-NPM) with or without plasmids expressing V5-tagged GLTSCR2 (V5-GLTSCR2) and His-ubiquitin. As shown in Figure3B, overexpression of GLTSCR2 promoted polyubiquitination of NPM. Next, to elucidate the mechanism of enhanced degradation of NPM by GLTSCR2, we determined whether GLTSCR2 altered the oligomerization status of NPM, as the oligomer form of NPM is the major and stable entity in HeLa cells and non-oligomeric NPM released from the nucleolus to the nucleoplasm is degraded quickly 22,23. HeLa cells were transfected with either pGFP or pGFP-GLT plasmid and lysed after treatment with 0.1% glutaraldehyde. Immunoblotting showed that overexpression of GLTSCR2 resulted in decrease in oligomer form of NPM, compared to overexpression of GFP alone (Fig.3C). Our results indicated that GLTSCR2 disrupted the oligomerization of NPM and enhanced the degradation of NPM through the proteasomal ubiquitination pathway.

### GLTSCR2 attenuates the increased transforming activity induced by NPM overexpression

**Figure 4:**
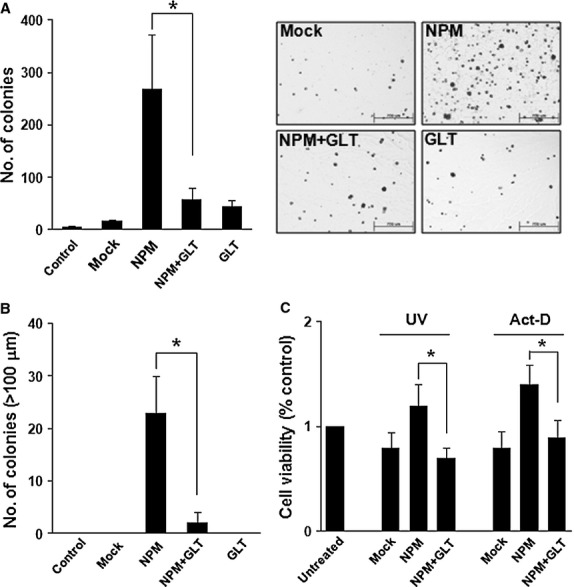
GLTSCR2 suppresses transforming activity and decreases apoptotic resistance induced by NPM. (A) HeLa cells were grouped into not transfected (control), singly transfected or cotransfected samples, as indicated. After 24 hrs of transfection, cells were plated at 10^4^ cells/60 mm dish in soft agar. Colony-forming assays were performed in soft agar and colony formation was evaluated 3 weeks after seeding. Data are shown as mean ± SD; *P < 0.05. Representative images are shown in the right panel. (B) Numbers of colonies more than 100 μm in diameter are shown. Data are presented as mean ± SD; *P < 0.05. (C) Cells were categorized as ‘A’, exposed to UV (50 J/m^2^) or ‘B’, treated with 50 μM Act-D for 24 hrs and evaluated using the MTT assay. Data are shown as fold-increase compared to control cells from three independent experiments (mean ± SD; *P < 0.05).

Nucleophosmin increases transforming activity and cell survival against apoptotic signals 24. Thus, we wanted to confirm whether NPM down-regulation by GLTSCR2 influenced survival and colony formation in soft agar by virtue of anchorage-independent growth of cervical cells. HeLa cells were singly transfected with NPM-expressing plasmid or cotransfected with plasmids expressing either NPM or GLTSCR2, and anchorage-independent growth assays were performed. As shown in Figure4A, NPM overexpression increased colony formation in soft agar as compared to mock-transfected cells. Interestingly, co-expression of GLTSCR2 significantly suppressed colony survival induced by NPM overexpression. In addition, cells cotransfected with NPM and GLTSCR2 formed smaller colonies than cells singly transfected with NPM (Fig.4B). Next, to elucidate whether GLTSCR2 restored cell survival induced by NPM overexpression, cells singly transfected with NPM or cotransfected with NPM and GLTSCR2 were exposed to UV or treated with actinomycin D (Act-D). Overexpression of NPM inhibited cell death induced by UV or Act-D, whereas co-expression of NPM and GLTSCR2 restored the sensitivity to UV or Act-D (Fig.4C). Our results demonstrated that GLTSCR2 expression attenuated the increased resistance to apoptosis and transforming activity induced by NPM overexpression.

### Expression levels of NPM is reciprocally associated with GLTSCR2 expression in cervical cancer tissues

**Figure 5:**
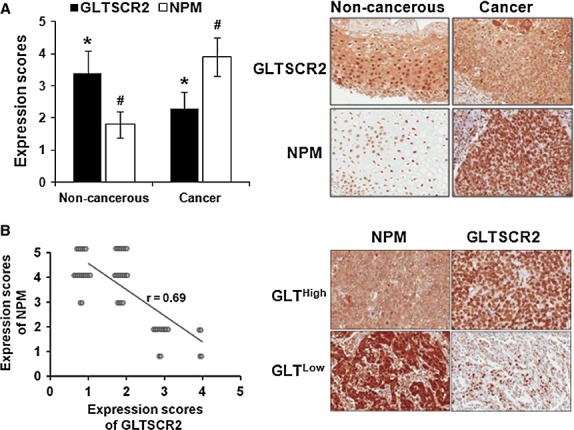
Mutually exclusive expression of GLTSCR2 and NPM in cervical cancer tissues. (A) The histogram shows mean immunohistochemical (IHC) scores (mean ± SD) of GLTSCR2 from non-cancerous and cervical cancer tissues. Representative IHC images are shown in the right panel (Original magnification ×100). (B) Spearman's rank correlation between NPM and GLTSCR2 scores. Representative IHC images are shown in the right panel (Original magnification ×100). *^,#^*P* < 0.05.

In this study, we demonstrated that GLTSCR2 promoted degradation of NPM protein in HeLa cells. However, it was unclear whether GLTSCR2 expression is down-regulated in cervical cancer tissues as compared to normal cervical epithelial tissues. To address this, we performed immunohistochemical staining for GLTSCR2 and NPM in 52 cases of squamous cell carcinomas of the uterine cervix and compared the expression levels of GLTSCR2 and NPM in cancer tissues to adjacent normal cervical tissue within same specimen. As shown in Figure5A, the expression score of GLTSCR2 was significantly lower in cancer tissues than in non-cancerous cervical tissues. In contrast, NPM expression was higher in cancer tissues than in non-cancerous tissues. Next, we determined whether NPM expression is associated with GLTSCR2 expression. As depicted in Figure5B, GLTSCR2 expression is reciprocally correlated with that of NPM in cervical cancer tissues (Spearman's correlation coefficient; *r* = −0.69, *P* < 0.01). Taken together, our results imply that cervical cancer tissues with low GLTSCR2 expression may have enhanced growth potentials *via* up-regulated NPM expression.

## Discussion

The nucleolar protein NPM plays a crucial role in oncogenesis 4,5. NPM, in the nucleolus, participates in ribosome biogenesis by regulating rRNA maturation 25 and traps ARF within the nucleolus to prevent p53 activation 26. Thus, overexpression of NPM promotes cell growth, inhibits cell death and accelerates cell cycle progression by promoting entry into the S-phase in the absence of p53 21. Consistent with these findings, NPM overexpression is found in several types of human cancer 5. Conversely, NPM plays a role in tumour suppression. Oncogenic stress induces nucleoplasmic translocation of NPM, which stabilizes p53 and inhibits cell cycle progression 10,11. In this regard, our results indicate that GLTSCR2 suppresses oncogenic activity induced by NPM through (*i*) the release of NPM from the nucleolus to the nucleoplasm and (*ii*) enhanced degradation of NPM.

Although the molecular mechanism for NPM translocation by GLTSCR2 is not clearly defined, one putative mechanism is the disruption of the oligomerization of NPM. NPM tends to form oligomers in cells *via* its N-terminal domain 27 and NPM oligomer is the major form *in vivo* 22. Recent studies demonstrate that the oligomer state of NPM modulates its various biological activities. For example, putative tumour suppressor, HLJ1 interacts with NPM and increases the oligomer form of NPM. Enforced accumulation of NPM oligomers by overexpression in HLJ1-expressing cells induced increased cellular migration, invasiveness and colony formation 28. Moreover, recent studies showed that blockade of endogenous NPM1 oligomerization by small molecular inhibitors or NPM peptides inhibits cell proliferation and induces apoptosis 29,30. These studies consistently showed that the oligomer form of NPM has cell proliferating activity, whereas disruption of oligomer inhibits proliferation activity. Here, we showed that GLTSCR2 overexpression disrupts the oligomeric form of NPM and inhibits NPM-induced cell proliferation and colony-forming activities.

Aberrant overexpression of NPM characterizes it as an oncoprotein. One of the causes of NPM overexpression in cancer is chromosomal translocation between the NPM gene and ALK tyrosine kinase (NPM/ALK) 31, retinoic acid receptor (NPM/RAR) 32 or MLF1 (NPM/MLF1) 33. Another genetic change in NPM during cancer includes mutation or loss of heterozygosity 34,35. However, the majority of patients with these genetic changes are confined to specific tumour types, such as haematologic malignancies. Little is known about the mechanism for NPM overexpression in solid tumours such as gastrointestinal 36,37, ovarian 38 and prostatic cancers 39. In the present study, we demonstrated that GLTSCR2 overexpression shortened the half-life of NPM to approximately 10 hrs, augmented polyubiquitination, and promoted the proteasomal degradation of NPM. In addition, we showed mutually exclusive expression of NPM and GLTSCR2 in cervical cancer tissues as well as frequent down-regulation of GLTSCR2 and up-regulation of NPM in human cervical cancer tissues. Our results imply that cervical cancer tissues with reduced GLTSCR2 expression have a chance for enhanced growth potential through up-regulated NPM expression and that GLTSCR2 might participate in suppression of tumourigenesis *via* NPM repression in cervical cancer.

In summary, we showed that the nucleolar protein GLTSCR2 triggered nucleoplasmic translocation of nucleolar NPM and down-regulated NPM by accelerated protein degradation through ubiquitination and the proteasomal pathway. This resulted in the inhibition of NPM-induced cell proliferative and transforming activities. Mutually exclusive expression of GLTSCR2 and NPM in cervical cancer tissues along with down-regulated GLTSCR2 expression as compared to normal cervical squamous epithelium corroborated our hypothesis that GLTSCR2 plays an important role in development and growth of cervical cancer cells.
